# Pioglitazone inhibits HIF-1α-dependent angiogenesis in rats by paracrine and direct effects on endothelial cells

**DOI:** 10.1007/s00109-013-1115-0

**Published:** 2014-01-10

**Authors:** Peter Dromparis, Gopinath Sutendra, Roxane Paulin, Spencer Proctor, Evangelos D. Michelakis, M. Sean McMurtry

**Affiliations:** Department of Medicine, University of Alberta, 8440 112th Street, Edmonton, AB, T6G 2B7 AB Canada

**Keywords:** Angiogenesis, Thiazolidinediones, Diabetes, Peripheral arterial disease, Mitochondria

## Abstract

**Abstract:**

Pioglitazone was associated with increased hazard for surgical or percutaneous lower extremity revascularization in patients with diabetes in a large clinical trial, but this clinical finding has not been adequately explored in animal models. We hypothesized that pioglitazone would decrease hypoxia-inducible factor 1α (HIF-1α)-dependent angiogenesis in rat ischemic hindlimb models by altering mitochondrial-derived signals supporting HIF-1α activation. We tested oral pioglitazone (10 mg/kg/day) versus placebo in two cohorts of rats with hindlimb ischemia (normal Sprague–Dawley rats and insulin-resistant JCR:La-cp rats), and evaluated direct and paracrine effects of pioglitazone on angiogenesis in vitro using human skeletal muscle and endothelial cells. Pioglitazone treatment was associated with reductions in limb perfusion at 2 weeks measured by contrast-enhanced ultrasound and Tc^99m^-Sestamibi SPECT-CT. Ischemic muscle capillary density was also reduced by pioglitazone. HIF-1α and vascular endothelial growth factor (VEGF) expression in ischemic muscle were also reduced by pioglitazone. In vitro, pioglitazone's effects on both skeletal muscle cells and microvascular endothelial cells were associated with a decrease in autocrine and paracrine angiogenesis measured by matrigel assay, decreased HIF-1α expression and activation, as well as increases in both mitochondrial reactive oxygen species and α-ketoglutarate, both mitochondria-derived signals which promote HIF-1α degradation. We conclude that pioglitazone is associated with decreased ischemic limb perfusion and capillary density in relevant rat models of hindlimb ischemia, and these effects are mediated by mitochondria-dependent reductions in HIF-1α-dependent angiogenesis.

**Key messages:**

- Pioglitazone inhibits angiogenesis in rats with and without insulin resistance.
- Pioglitazone inhibits HIF-1α by inhibiting mitochondrial stabilization of HIF-1.
- Pioglitazone inhibits both autocrine and paracrine angiogenesis.
- Inhibition of angiogenesis may explain unexpected results of a pioglitazone human clinical trial.

**Electronic supplementary material:**

The online version of this article (doi:10.1007/s00109-013-1115-0) contains supplementary material, which is available to authorized users.

## Introduction

Peripheral artery disease (PAD) is a common disorder due to atherosclerosis of the leg arteries [1]. Diabetes is a risk factor for PAD [2], and in patients with diabetes, concomitant PAD is a risk factor for lower extremity amputation [3]. Both diabetes [4, 5] and PAD [6] are associated with adverse cardiovascular outcomes, including death, myocardial infarction, and stroke. Pioglitazone is a peroxisome-proliferator-activated receptor-γ (PPARγ) ligand that was developed as oral hypoglycemic therapies for type 2 diabetes [7]. The PROactive trial, a randomized trial of pioglitazone versus placebo in 5238 patients with diabetes [8], unexpectedly showed that pioglitazone was associated with increased hazard for surgical or percutaneous lower extremity revascularization (hazard ratio 1.69, 95 % CI 1.153, 2.484) [9]. Moreover, those with PAD did not benefit from pioglitazone with respect to other endpoints like cardiovascular death or acute coronary syndrome [9]. Though other randomized clinical trials of pioglitazone support that pioglitazone use by patients with diabetes lowers risk for death, myocardial infarction, and stroke, these other studies did not evaluate limb-related outcomes nor include patients with PAD as a subgroup [10]. The mechanisms for adverse effects in subjects with PAD are not known.

The mechanism of action of thiazolidinediones is incompletely understood, but includes PPARγ-dependent tissue-specific alterations in fatty acid uptake and storage, with concomitant improvements in peripheral tissue insulin sensitivity and utilization of glucose [7]. Despite evidence that these drugs have direct effects on endothelial cells, leading to decreased angiogenesis in vitro [11], the possibility that this may explain adverse effects in PAD patients has not been adequately studied in appropriate in vivo models.

We speculated that pioglitazone's effects on muscle mitochondria may inhibit HIF-1α, which could decrease expression of proangiogenic factors, suppressing angiogenesis in a paracrine manner. We have recently described a model by which mitochondria-derived signals [including mitochondria-derived reactive oxygen species (mROS) and α-ketoglutarate (αKG), a diffusible Krebs' cycle product] can directly regulate HIF-1α (which is redox-sensitive and requires αKG in its hydroxylation-driven destabilization) in cancer cells, leading to decreased paracrine angiogenic signaling in the tumor [12]. We speculated that a similar mechanism may take place in the skeletal muscle, particularly since there is some evidence that thiazolidinediones can affect mitochondria function via PPARγ (i.e., mitochondrial biogenesis) or by direct interaction to mitochondrial proteins, like complex I [13], a major site for mROS production.

We tested this hypothesis in a set of in vivo experiments involving healthy (Sprague–Dawley) and insulin-resistant rats (JCR:LA-cp) [14] and in vitro techniques to explore both paracrine as well as direct effects of pioglitazone in angiogenesis using both human skeletal muscle and endothelial cells.

## Materials and methods

### Rat hindlimb ischemia model

All experiments were conducted with the approval of the Animal Care and Use Committee of the University of Alberta. Adult male Sprague–Dawley (*n* = 22) and obese JCR:LA-cp insulin-resistant (*n* = 20) rats [15] were sequentially numbered, anesthetized with isofluorane anesthesia and placed on a heated surgical table. Using sterile technique, the left common iliac artery and left superficial femoral artery were each ligated twice with 3.0 silk suture and cut between the ligatures. The skin was close in one layer with 5.0 prolene suture. After recovery, animals were randomized with allocation concealment to receive either oral pioglitazone (10 mg/kg per day; Takeda Pharmaceuticals, Deerfield, IL) or placebo. Only one technician knew the randomization key, and investigators blinded to treatment allocation completed all subsequent experiments. A separate group of Sprague–Dawley rats were used in preliminary experiments to validate the model, including in vivo and ex vivo angiography and Doppler ultrasound, as well as to validate the Tc^99m^-sestamibi perfusion imaging technique. An AM glucose measurement was performed at the end of the protocol in the JCR:LA-cp rats to confirm a hyperglycemic state (Omron Healthcare, Scarborough, ON).

### Tc^99m^ sestamibi SPECT-CT imaging

Anesthetized rats were placed in the bed of a PET-SPECT-CT (Gamma Medica, Northridge, CA). A dose of 2.0 mCu of Tc^99m^-labeled sestamibi was injected via a central venous cannula. After 15 min, a CT scan and SPECT images were obtained. Three-dimensional CT and SPECT datasets were fused and analyzed using Amira Software (Visage Imaging Incorporated, San Diego, CA). Signal within the gastrocnemius muscle was identified and quantified based on segmentation of a region of interest based on the three-dimensional CT datasets.

### Contrast-enhanced ultrasound perfusion imaging

Anesthetized rats were secured on the heated table of a Vevo 770 rodent ultrasound machine (Visualsonics, Toronto, ON). The right and left calves were denuded of hair using a chemical depilatory. Using a 707B probe, a B-mode image of either the right or left calf muscle was obtained. The acquisition was triggered, and after 5 s (30 frames) a bolus of 3.5 × 10^8^ microbubbles (Visualsonics, Toronto, ON) was injected into a central venous cannula. A total of 425 frames of data were collected. After 10 min of exposing the rodent to an inhaled FiO_2_ of 100 % (to destroy residual bubbles), the procedure was repeated to image the contralateral limb. Perfusion curves were generated and curve-fit offline using the Vevo analysis software (Visualsonics Toronto, ON).

### Fluorescent microspheres

Ex vivo calf muscle perfusion was measured as previously described [16]. Briefly, a bolus of 3.6 × 10^6^ 10 μm diameter microspheres were administered to an anesthetized animal via central venous cannula and allowed to circulate for 15 min prior to euthanasia. Muscle tissues were excised and digested in ethanolic KOH. Microspheres were sedimented by centrifugation, fluorescent dye was extracted, and fluorescence was measured with a GEMINI-XS fluorimeter (Molecular Devices, Sunnyvale, CA).

### Cell culture

Human microvascular endothelial cells (hMVECs; Cascade Biologics, Portland, OR) and human skeletal muscle cells (hSkMCs; Promocell, Heidelberg, Germany) were cultured in M131medium (Invitrogen, Carlsbad, CA) and Promocell C-23060 media (Promocell, Heidelberg, Germany), respectively. Up to passage, six cells were used. For the autocrine matrigel studies, hMVECs were cultured in normoxia (pO_2_ 120 mmHg), hypoxia (pO_2_ 40 mmHg), or hypoxia plus pioglitazone at 1, 10, or 25 μg/ml for 48 h.

### Matrigel assays

Matrigel was prepared in a 12-well plate per manufacturer's instructions (BDBiosciences, Mississauga, ON), and 50,000 hMVECs/well were applied. hMVECs were then cultured in either normoxia or hypoxia for 4 h, and images were obtained (5 images/well ×40 magnification). Tubule length and number of completely enclosed structures per image were measured using Image Pro-Plus software version 6.2.0 (Media Cybernetics). Five wells on each of five plates were evaluated per group. In the Paracine Matrigel Assay, a Boyden chamber was used. Briefly, hSkMCs were cultured in normoxia (pO_2_ 120 mmHg), hypoxia (pO_2_ 40 mmHg), or hypoxia plus pioglitazone (20 μg/ml) for 48 h and washed to remove all medium and pioglitazone. Matrigel was prepared in a similar manner and hMVECs, previously cultured at pO_2_ 120 mmHg, were applied to the matrigel. The washed hSkMCs were placed in the top insert of a 12-well 0.4 μm Boyden chamber (Corning, Corning, NY), and placed in contact with medium over the hMVEC cells. The cells were incubated at pO_2_ 120 mmHg for 4 h. Images were obtained and data was analyzed as described above (see Fig. 3a).

### Confocal microscopy and lectin perfusion

All imaging was performed with a Zeiss LSM 510 Confocal microscope (Carl Zeiss, Toronto, ON). Live cell imaging of mitochondrial membrane potential (TMRM; Invitrogen, Burlington, ON) and mitochondrial ROS (Mitosox; Invitrogen, Burlington, ON) were imaged as previously described. Primary antibodies used include VEGF (1:100; Santa Cruz Biotechnologies), HIF-1a (1:100; Abcam, San Francisco, CA), vWF (1:200; Abcam, San Francisco, CA, San Francisco, CA), SMA (1:100; Abcam, San Francisco, CA). For imaging of hypoxic cells, stains were applied onto cells within the hypoxic incubator, and imaged within in a custom chamber suffused with 5 % oxygen gas to preserve hypoxic conditions. Lectin fluorescein ricinus communis agglutinin I (5 mg) (Vector Laboratories, Inc, Burlingame, CA) was injected via a central venous cannula for 5 min prior to sacrifice, gastrocnemius isolation and flash freezing. For semi-quantification, randomly selected fields were evaluated and regions of interest were semi-quantified in arbitrary fluorescence units using the Zeiss LSM Image Examiner software version 3.0.2.70 (Carl Zeiss, Toronto, ON). Confocal laser settings were adjusted for background signals from a negative control (secondary antibody only) would register an intensity of zero. For HIF-1α, only nuclear signals were included in the regions of interest.

#### Assessment of arteriogenesis

Five randomly selected sections of both the right and the left gastrocnemius muscle from five JCR:LA-*cp* rats from each treatment group were quantified. Lectin fluorescein ricinus communis agglutinin I was injected (see above) prior to sacrifice, and ex vivo the sections were stained with vWF and SMA. The number of muscularized (SMA positive) arterioles per high-power field was measured, with three to four high-power fields per section evaluated per section.

### Western blot and qRT-PCR

Tissues were collected and Western blotting was performed with standard technique using 25 μg of protein per sample. The films were digitized and quantified using 1D Image Analysis Software (Kodak, Rochester, NY). Expression was normalized to actin to correct for loading differences. For qRT-PCR, we used commercially available primers in an Applied Biosystems qRT-PCR machine (ABI PRISM 7700) as previously described [17].

### α-ketoglutarate assay

hSkMCs were cultured in normoxia, hypoxia or hypoxia + 20μg/ml pioglitazone for 48 h. The α-ketoglutarate (αKG) levels were measured as described in a commercially available spectro-photometric αKG Assay Kit (BioVision, Mountain View, CA, USA). hSKMCs cells were grown to confluency in a T75 flask. Cells were then harvested, lysed, and protein concentration was adjusted to equal levels between groups. The αKG levels were measured by measuring OD at 570 nm after the kit-based reaction was completed as previously described [18].

### Complex I activity assay

Complex I activity was measured with a complex I activity kit (MitoSciences, Eugene, OR). The hSkMCs were cultured in normoxia, hypoxia or hypoxia + 20 μg/ml pioglitazone for 48 h. Protein was collected after cells were homogenized. Protein (50 μL of 1 μg/mL) was placed in a 96-well dish and incubated with the dipstick containing an antibody to Complex I and then incubated in activity buffer. Complex I activity was measured by intensity of band using a flat top scanner.

### Statistical Analysis

Statistics was performed on SPSS 18.0 Software (Somers, NY). Values are expressed as mean ± SEM. Comparisons between two groups for in vivo experiments were made using Mann-Whitney *U* test. For in vitro data, a Mann–Whitney *U* test was used to compare two groups or Kruskal-Wallis test was performed to compare among two or more groups. Significance was considered at *p* < 0.05.

## Results

### Model validation

We found that our hindlimb ischemia model was feasible with no postoperative mortality. CT angiography (supplemental Figure 1a, c) and Doppler ultrasound (supplemental Figure 1b) confirmed successful interruption of the large arteries of the left leg with reduced distal flow. This model, in both Sprague–Dawley and JCR:LA-cp rats, recapitulated features of human limb ischemia, including superficial lower extremity ulcers (supplemental Figure 1d). Standardized morning blood glucose measurements performed on the JCR:LA-cp rats found average blood glucose of 22.4 ± 2.5 mmol/l in placebo-treated rats, and 12.5 ± 1.2 mmol/l in pioglitazone-treated rats. In a separate set of Sprague–Dawley rats with hindlimb ischemia, we found that the ischemic reserve (ischemic leg perfusion divided by non-ischemic leg perfusion) measured by Tc^99m^-Sestamibi SPECT-CT imaging significantly correlated with that measured by fluorescent microspheres (the gold standard method) with a Pearson coefficient 0.798, validating Tc^99m^-Sestamibi SPECT-CT as an assay of perfusion in our models.

### Pioglitazone decreased ischemic limb perfusion

**Fig. 1:**
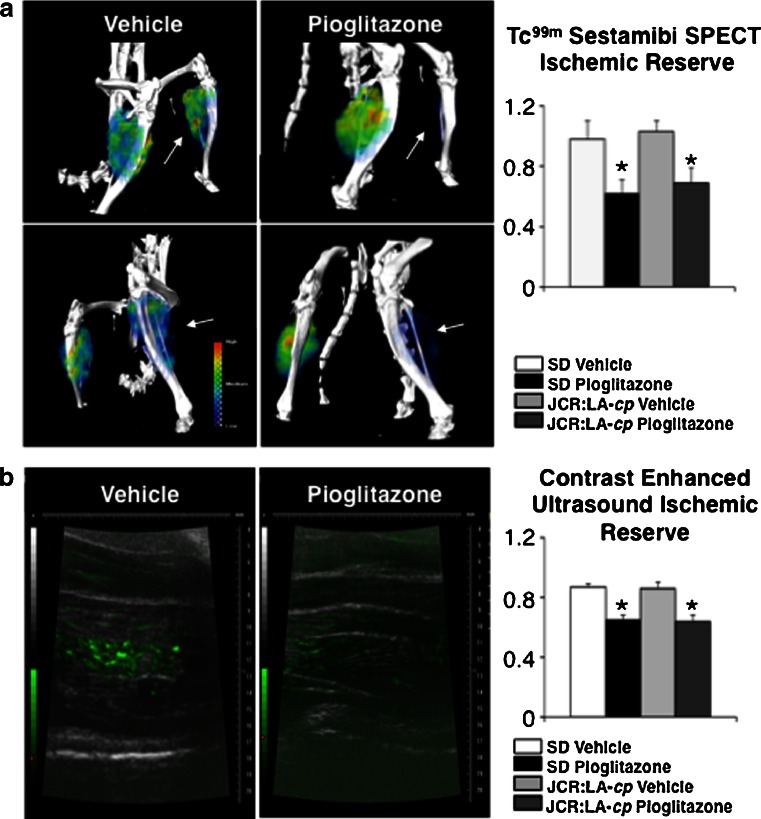
Pioglitazone is associated with reduced gastrocnemius perfusion in the hind limb ischemia model. **a** Representative SPECT-CT images of the gastrocnemius muscle after Tc^99m^-Sestamibi injection. Pioglitazone decreases perfusion in both diabetic and non-diabetic models. Values are represented as mean ± SEM of the ischemic reserve (the ratio of the signal in the ligated/non-ligated leg) (*n* = 20 animals/group. *p < 0.05 vs. respective placebo-treated control). **b** Representative contrast-enhanced ultrasound images of the gastrocnemius muscle after micro-bubble injection. Pioglitazone decreases perfusion in both diabetic and non-diabetic models. Values are represented as ischemic reserve mean ± SEM (the ratio of the signal in the ligated/non-ligated leg) (*n* = 20 animals/group. *p < 0.05 vs. respective placebo-treated control)

We assessed in vivo limb perfusion bilaterally after 2 weeks of either pioglitazone or vehicle treatment using two techniques, including Tc^99m^-Sestamibi SPECT-CT (validated by us) and contrast-enhanced ultrasound (previously validated by others [19]). We found significantly reduced perfusion with both techniques in pioglitazone-treated Sprague–Dawley and JCR:LA-cp rats, compared with vehicle-treated controls. In Sprague–Dawley rats, Tc^99m^-Sestamibi SPECT-CT ischemic reserve was 0.98 ± 0.12 in vehicle-treated rats compared with 0.62 ± 0.09 in pioglitazone-treated rats (Fig. 1a). In JCR:LA-cp rats, the Tc^99m^-Sestamibi SPECT-CT measured ischemic reserve was 1.03 ± 0.07 in the vehicle-treated rats, compared with 0.69 ± 0.10 in the pioglitazone-treated rats (Fig. 1a). Contrast-enhanced ultrasound measurements of ischemic reserve were similar to the Tc^99m^-Sestamibi SPECT-CT results. In Sprague–Dawley rats, contrast-enhanced ultrasound measured ischemic reserve was 0.87 ± 0.02 in vehicle-treated rats, compared with 0.65 ± 0.03 in pioglitazone-treated rats (Fig. 1b). In JCR:LA-cp rats, contrast-enhanced ultrasound measured ischemic reserve was 0.86 ± 0.03 in the vehicle-treated rats, compared with 0.64 ± 0.04 in the pioglitazone-treated rats (Fig. 1b).

### Pioglitazone decreased ischemic limb capillary density

**Fig. 2:**
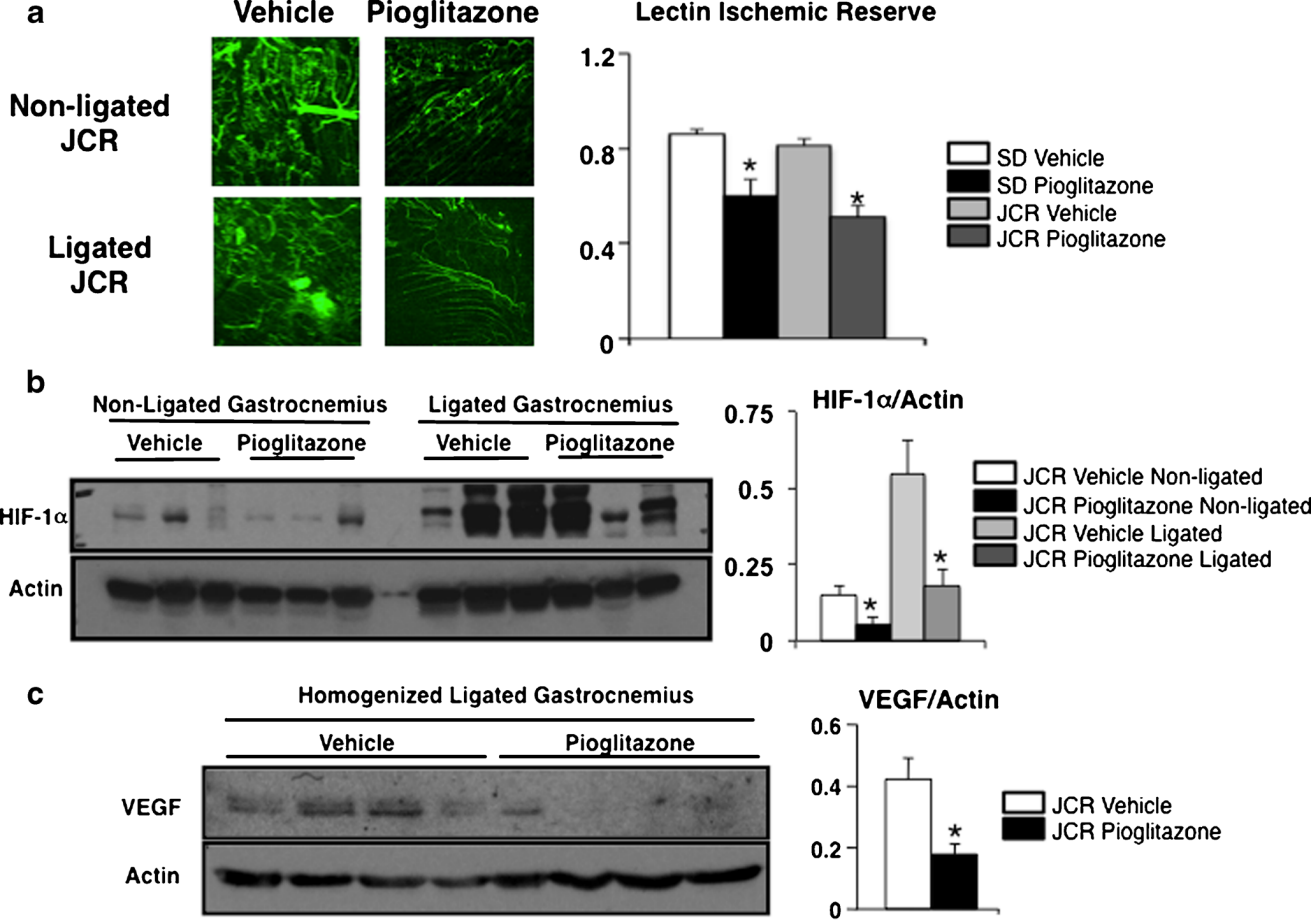
Pioglitazone is associated with reduced gastrocnemius lectin perfusion in vivo. **a** Representative confocal images of the gastrocnemius muscle of vehicle- (*left*) and pioglitazone-treated rats perfused with lectin (*green*) immediately prior to sacrifice. Pioglitazone decreases the lectin perfusion (*left*) in both diabetic and non-diabetic models compared to vehicle controls. Values are represented as mean ± SEM of the ischemic reserve (the ratio of the signal in the ligated/non-ligated leg) (*p < 0.05 vs. respective placebo-treated control group). *Bar* 200 μm. **b** Western blot showing reduced HIF-1α expression in both non-ligated and ligated limbs exposed to pioglitazone (*p < 0.05 vs. vehicle-treated group). Three different blots, each with samples from three different rats, were quantified. Each lane represents muscle tissue from one animal. **c** Western blot showing reduced VEGF expression in the gastrocnemius muscles with ligated vessels in pioglitazone-treated animals compared to vehicle-treated animals (*p < 0.05 vs. vehicle-treated group)

We assessed capillary density in gastrocnemius muscles ex vivo by confocal fluorescence microscopy for fluorescent lectin, perfused prior to euthanasia, and by immunohistochemistry for the endothelial cell marker von Willebrand factor (vWF). We found reduced capillary density with both endothelial markers in pioglitazone-treated Sprague–Dawley and JCR:LA-cp rats compared with relevant controls (Fig. 2a and supplemental Figure 2). In Sprague–Dawley rats, the ratio of lectin signal in the ligated versus non-ligated limb was 0.86 ± 0.02 in vehicle-treated rats compared with 0.60 ± 0.07 in pioglitazone-treated rats. In JCR:LA-cp rats, the ratio of lectin signal in the ligated versus non-ligated limb was 0.81 ± 0.03 in the vehicle-treated rats, compared with 0.51 ± 0.05 in the pioglitazone-treated rats. In addition, we stained muscle tissue with vWF and smooth muscle actin, and counted muscularized arterioles in the ischemic limb adductor muscles of the JCR:LA-cp rats as an index of arteriogenesis. We found that there were increased numbers of muscularized arterioles in the ischemic limbs compared with the non-ischemic limbs in both placebo-treated (4.0 ± 0.3 versus 2.6 ± 0.2 muscularized arterioles per field) and pioglitazone-treated (3.3 ± 0.3 versus 2.2 ± 0.2 muscularized arterioles per field) JCR:LA-cp, consistent with enhanced arteriogenesis in the ligated (ischemic) limb (Supplemental Figure 3). However, the number of muscularized arterioles was not reduced in the pioglitazone-treated ischemic limbs compared with the placebo-treated ischemic limbs (3.3 ± 0.3 versus 4.0 ± 0.3, *p* = 0.15).

### Pioglitazone reduced expression of VEGF in ischemic gastrocnemius muscle

We evaluated in vivo expression of HIF-1α and VEGF by performing Western blots on homogenized ischemic gastrocnemius muscles from placebo and pioglitazone-treated JCR:LA-cp rats. We found that both HIF-1α and VEGF protein expression was reduced in ischemic muscle tissue in pioglitazone-treated rats compared with placebo-treated rats (Fig. 2b, c).

### Pioglitazone inhibits angiogenesis through autocrine and paracrine mechanisms

**Fig. 3:**
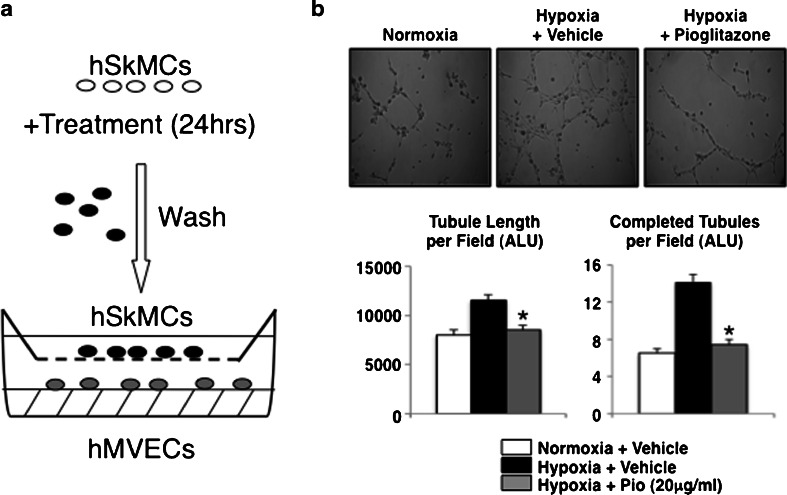
Pioglitazone decreases paracrine angiogenic signaling in vitro. **a** Schematic of the experimental procedure. hSkMCs were pretreated in normoxic, hypoxic, or hypoxic + pioglitazone conditions for 24 h. Cells were collected, washed to remove any residual pioglitazone and plated in the top of a Boyden chamber with MVECs on a matrigel assay in normoxic conditions. A porous membrane that only allows diffusion of secreted factors separated the hSKMCs and MVECs. **b** Representative images hMVECs co-cultured with pretreated hSkMCs on a Boyden chamber matrigel assay. Pioglitazone blocks hypoxia-induce angiogenesis in both total tubule length per field (*left*) and completed tubule structures per field (*right*) in a dose-dependent manner (*n* = 5 images/well, 5 wells/group/experiment, 3 experiments. *p < 0.05 vs. hypoxia vehicle)

To specifically address whether pioglitazone inhibits angiogenesis by a paracrine effect via acting on skeletal muscle, we used a bioassay in which we exposed hMVECs to hSkMCs pretreated with normoxic media + vehicle, hypoxic media + vehicle or hypoxic media + pioglitazone, for 48 h. The pretreated hSkMCs were washed and placed on the top section of a Boyden chamber, so that only products actively secreted from the muscle cells would be released in the bottom chamber, avoiding the confounding from the drugs used in the pretreatment (Fig. 3a). In the bottom chamber, we placed hMVEC in matrigel. Exposure to hSkMCs pretreated with hypoxia + vehicle led to increased hMVEC tubule formation (assessed by both tubule length and number of completed tubules), compared to hSkMCs pretreated with normoxia + vehicle (Fig. 3b), consistent with a hypoxia-driven paracrine angiogenic signaling. This increase in angiogenesis was completely inhibited during the exposure to hSkMCs pretreated with vehicle + pioglitazone. This suggested that despite the presence of hypoxia, pioglitazone inhibited the proangiogenic response in skeletal muscle, leading to a decrease in the muscle production of secreted proangiogenic factors, consistent with the decrease production of VEGF in the muscle tissues in vivo (see Fig. 2c). To confirm a direct inhibition of angiogenesis by pioglitazone on endothelial cells, we also studied hMVECs exposed directly to hypoxia + pioglitazone or vehicle. Hypoxia increased in vitro angiogenesis, assessed by both total tubule length and the number of complete tubules per field, and pioglitazone decreased tubule formation in a dose-dependent manner (supplemental Figure 4).

### Pioglitazone inhibits HIF-1α activation in hypoxia hSkMCs

**Fig. 4:**
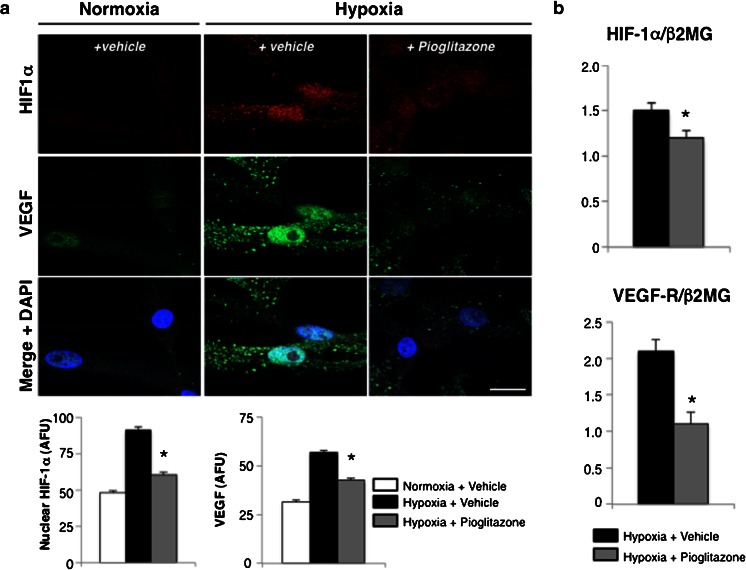
Pioglitazone inhibits HIF-1α in hSkMCs. **a** Representative immunofluorescence confocal images of hSkMCs in normoxic, hypoxic, or hypoxic plus pioglitazone conditions and stained with HIF-1α (*red*), VEGF (*green*), and the nuclear stain DAPI (*blue*). Pioglitazone reduces nuclear (active) HIF-1α and VEGF expression in hypoxic hSkMCs (*p < 0.05 vs. hypoxia vehicle). *Bar* 20 μm. **b** mRNA expression of HIF-1α target genes, HIF-1α and VEGF-R, in hSkMCs exposed to hypoxia plus vehicle or pioglitazone. Pioglitazone reduces HIF-1α and VEGF-R mRNA (*n* = 3 experiments. *p < 0.05 vs. hypoxia vehicle)

To determine whether the impaired paracrine-mediated angiogenesis involved suppression of HIF-1α, we performed immunofluorescence studies to determine the cellular location of HIF-1α in hSkMCs. HIF-1α is active as a transcription factor when localized in the nucleus. Hypoxia induced robust increases in HIF-1α nuclear localization, which was reversed with pioglitazone (Fig. 4a). In parallel to the changes in HIF-1α nuclear expression, there was increased VEGF expression after exposure to hypoxia, which was decreased by pioglitazone. By using multiple staining technique, we showed that VEGF expression increased in the same cells in which nuclear HIF-1α expression increased. Pioglitazone also reduced overall HIF-1α and VEGF receptor mRNA expression levels (Fig. 4b), measured by qRT-PCR.

### Pioglitazone increases mitochondrial signaling in hypoxic hSkMCs

**Fig. 5:**
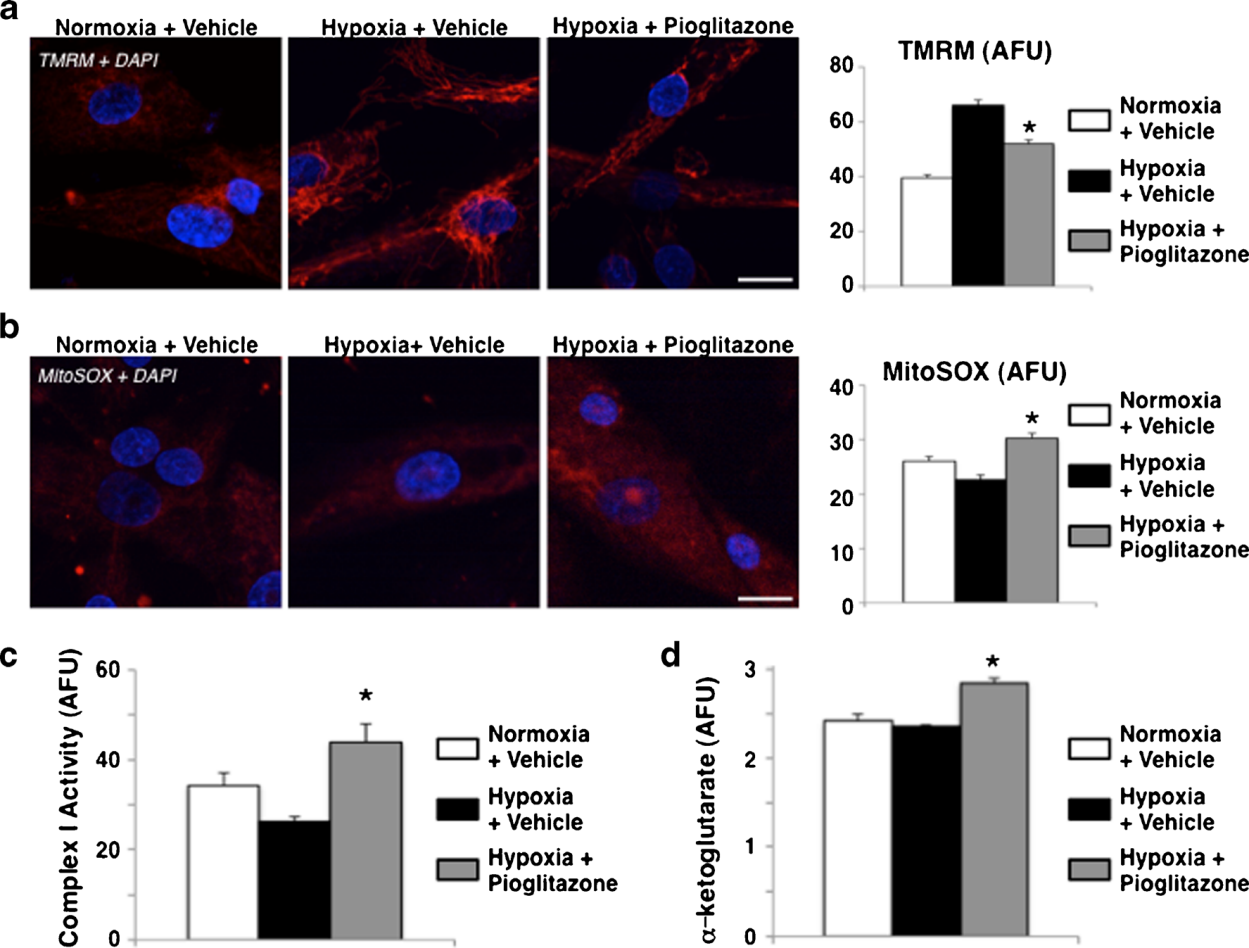
Pioglitazone enhances mitochondrial activity in hSkMCs. **a** Representative confocal images of hSkMCs exposed on normoxia, hypoxia, or hypoxia plus pioglitazone and stained with TMRM (*red*, ΔΨm marker) and DAPI (*blue*, nuclear marker). Hypoxia increases ΔΨm, which is partially blocked with pioglitazone (*n* = 40–50 cells/group/experiment, 3 experiments. *p < 0.05 vs. hypoxia vehicle). *Bar* 20 μm. **b** Representative confocal images of hSkMCs exposed on normoxia, hypoxia, or hypoxia plus pioglitazone and stained with MitoSOX (*red*, mROS indicator) and DAPI (*blue*). Hypoxia suppresses mROS, which is restored with pioglitazone (*n* = 40–50 cells/group/experiment, 3 experiments. *p < 0.05 vs. hypoxia vehicle). *Bar* 20 μm. **c** Complex I activity in hSkMCs exposed on normoxia, hypoxia, or hypoxia plus pioglitazone. Hypoxia reduces complex I activity, which is restored with pioglitazone treatment (*n* = 4 wells/group. *p < 0.05 vs. hypoxia vehicle). **d** αKG levels in hSkMCs exposed on normoxia, hypoxia, or hypoxia plus pioglitazone. Pioglitazone treatment increases αKG levels in hSkMCs (*n* = 5 wells/group. *p < 0.05 vs. hypoxia vehicle)

To determine whether pioglitazone-induced inhibition of angiogenesis involved changes in mitochondrial signaling, we measured mitochondrial membrane potential (ΔΨm), which links metabolic and oxidative functioning of the mitochondria. Since the ΔΨm is produced by efflux of H^+^ from the inner membrane, which is directly related to the flux of electrons through the electron transport chain (respiration) and the production of mROS, it is often used as a surrogate of mitochondrial function. Although the mechanism is complex and not entirely clear, hypoxia or functional mitochondrial suppression that is found in cancer cells or proliferating vascular cells, is associated with an increase in ΔΨm [12, 17, 20, 21]. In hSkMCs, hypoxia predictably increased ΔΨm (measured by the mitochondria specific voltage sensitive dye TMRM), but this was partially reversed by pioglitazone (Fig. 5a). Since HIF-1α expression and therefore function is regulated by α-KG and redox signals, we assessed mitochondrial production of these signaling molecules. mROS production, measured by the mitochondrial specific and redox-sensitive dye MitoSOX, was reduced (Fig. 5b). Similarly, α-KG was increased in pioglitazone-treated hypoxic hSkMCs (Fig. 5c). Complex I of the electron transport chain is the major producer of mROS, along with complex III. There is some evidence that pioglitazone may interact directly with Complex I [13]. Pioglitazone reversed a hypoxia-induced reduction in complex I activity (Fig. 5d). This increase in Complex I activity is compatible with pioglitazone's effect in increasing mitochondrial function and may explain both its effect on ΔΨm and mROS.

## Discussion

Our data show that pioglitazone is associated with impaired angiogenesis in vitro and in vivo in Sprague–Dawley and JCR:LA-cp insulin-resistant rats. Our findings are clinically relevant, in that impaired angiogenesis might account for an unexpected finding of the PROactive trial, which found that pioglitazone was associated with increased rates of lower extremity revascularization in the 1,274 subjects with concomitant PAD [9]. Inhibition of angiogenesis in ischemic limbs by pioglitazone, either by direct effects on endothelial cells or by a paracrine mechanism due to effects on skeletal muscle, may explain the increased hazard for surgical or percutaneous lower extremity revascularization in the pioglitazone treatment arm. In addition, observational studies of drug safety have also demonstrated an association between pioglitazone and increased rates of bone fractures [22]. Angiogenesis is well established as a key mechanism of bone healing and health [23], and pioglitazone-related impairments of angiogenesis might explain this treatment complication as well.

**Fig. 6:**
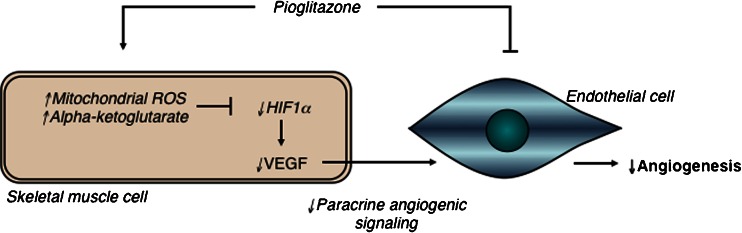
A proposed mechanism through which pioglitazone inhibits angiogenesis. Pioglitazone enhances mitochondrial activity in skeletal muscle tissue. This results in increased mitochondrial-derived signals—αKG and mROS—which can inhibit HIF-1α signaling. This disrupts skeletal muscle-derived angiogenic signals that act on neighboring endothelial cells, reducing vessel formation. This mechanism may run in parallel with direct antiangiogenic effects on endothelial cells, inhibiting angiogenesis through two mechanisms

In addition to demonstrating a direct antiangiogenic effect of pioglitazone on endothelial cells, we demonstrated that pioglitazone inhibits angiogenesis in a paracrine manner by inducing changes in mitochondrial function within skeletal muscle cells that reduce HIF-1α activation (Fig. 6). This is important since the effects of this class of drugs are thought to be due to their effects on skeletal muscle, a major site of insulin resistance in patients with metabolic syndrome and type II diabetes. These observations are analogous to our groups' novel demonstration of mitochondrial-dependent stabilization of HIF-1α within cancer cells that supports tumor angiogenesis [12]. These findings together suggest that this mitochondria-dependent regulation of HIF-1α may be relevant to angiogenesis within many organs and tissues, and that targeting mitochondrial function with metabolic modulating drugs could be a useful therapeutic strategy for many conditions linked with too much or too little angiogenesis. Our data on the potential direct effects of pioglitazone on endothelial cells are also in keeping with previous studies using in vitro and in vivo angiogenesis models [11, 24–28]. However, a proangiogenic effect of pioglitazone has been reported in adipose tissue [29], at least raising the possibility that effects of pioglitazone might be in part tissue-specific.

More data are required to evaluate the mechanism by which pioglitazone induces changes in mitochondrial function. The classical mechanism of action for PPARγ ligands like pioglitazone involves hetero-dimerization of PPARγ with the retinoid X receptor, specific DNA binding to PPAR response elements, and alteration of gene expression [30]. While alterations in gene expression induced in a PPARγ-dependent way could explain our results, direct and PPARγ-independent effects of pioglitazone on mitochondria, such as effects on Complex I activity [31, 32], could be responsible. Whether other thiazolidinedione drugs, like rosiglitazone, might impair angiogenesis in a similar manner was not directly tested by our data. However, though there are discordant results in the published literature, rosiglitazone has been associated with impaired angiogenesis by some investigators[24, 26], and it is plausible to speculate that other PPARγ ligands, like rosiglitazone, could have similar negative effects on angiogenesis.

Strengths of our work include using two rat models, including normal Sprague–Dawley rats and insulin-resistant JCR:LA-cp rats in a model system in which each ischemic limb is compared to its non-ischemic contralateral limb, minimizing variability. The insulin-resistant JCR:LA-cp rats are a good model for human type II diabetes [15] in that they are insulin resistant, obese, and prone to atherosclerosis [33]. We also used human cell lines for our in vitro studies. While there are limitations with any models of human disease, the use of JCR:LA-cp rats and human cell lines increases the likelihood our findings reflect biology within diabetic patients. Unlike many studies of hindlimb ischemia, we used two measures of perfusion, including contrast ultrasound and Tc^99m^-sestamibi SPECT imaging. The results from these two measures of limb perfusion were concordant, though the recovery after ischemic injury measured by contrast ultrasound was slightly lower than that measured by Tc^99m^-sestamibi SPECT, suggesting with minor differences in these techniques. Using these two measures together, complementing the in vivo findings with the ex vivo analysis of blood vessel density, and using randomization with allocation concealment and investigator blinding support, all support the veracity of our findings.

On the other hand, our data are in conflict with studies performed by Huang et al. [34] and Nagahama et al. [35], who found increased neovascularization in diabetic mice treated with oral pioglitazone (Huang et al. [34]) and non-diabetic mice treated with intramuscular injection of nanoparticles containing pioglitazone (Nagaham et al. [35]), respectively. There are methodological differences between our study and these two studies, however, with these studies having used different species, different drug doses and delivery route, more limited methods of assessing in vivo vascular perfusion, and apparent lack of randomization and investigator blinding.

We conclude that pioglitazone is associated with impaired angiogenesis in rats with hindlimb ischemia, in part due to direct effects on skeletal muscle mitochondria and due to mitochondria-dependent reductions in paracrine HIF-1α signaling. Reduced angiogenesis in ischemic tissues might explain clinical reports of adverse effects associated with pioglitazone.

## Electronic supplementary material

Below is the link to the electronic supplementary material.

ESM 1 (DOCX 424 kb)
