# Supplementary material for: Pioglitazone inhibits HIF-1α-dependent angiogenesis in rats by paracrine and direct effects on endothelial cells
Source: J Mol Med (Berl). 2014 Jan 10;92(5):497–507. doi: 10.1007/s00109-013-1115-0 (PMC3989538; doi:10.1007/s00109-013-1115-0)
Supplement: Supplementary file 1 — (DOCX 424 kb) [file 109_2013_1115_MOESM1_ESM.docx]

**Journal of Molecular Medicine 2014**

**Supplemantary Figures**

**Pioglitazone inhibits HIF-1α dependent angiogenesis in rats by paracrine and direct effects on endothelial cells**

Peter Dromparis, Gopinath Sutendra, Roxane Paulin, Spencer Proctor, Evangelos D. Michelakis, M. Sean McMurtry

**********


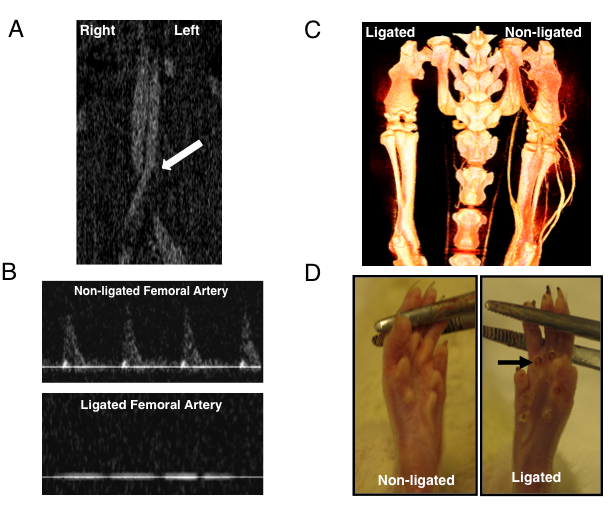
**Supplemental Figure 1. Model validation.** In vivo contrast enhanced CT angiogram 1 day after left common iliac and left femoral artery ligation shows lack of flow into the left iliac system (white arrow, Panel A). Pulse wave Doppler ultrasound of the right and left femoral artery 1 day after left common iliac and left femoral artery ligation confirm absence of flow in the left femoral artery (Panel B). An ex vivo barium angiogram 2 days after left common iliac and left femoral artery ligation illustrates lack of contrast enhancement of the named artery of the ligated left leg (Panel C, viewed from behind). After two weeks of limb ischemia, the ischemic limb frequently develops superficial lower extremity ulcers (Panel D), suggestive of severe limb ischemia.


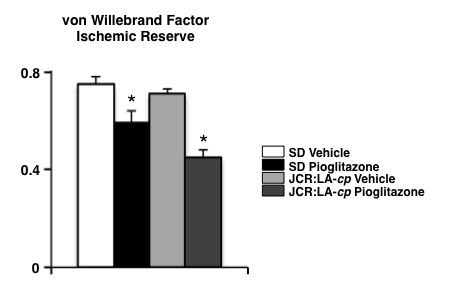


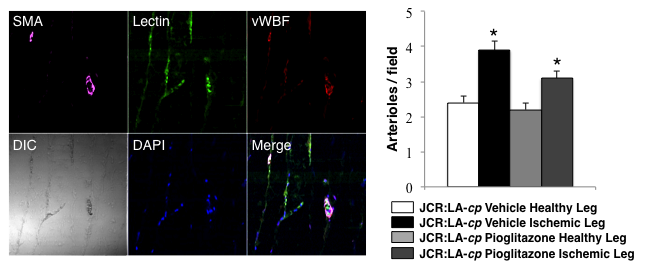
**Supplement Figure 2. Pioglitazone is associated with reduced gastrocnemius vessel density in vivo.** Pioglitazone decreases staining of the endothelial marker von Willebrand Factor (vWF) in the ischemic gastrocnemius muscle in both diabetic and non-diabetic models. Values are represented as mean ± SEM of the ischemic reserve (the ratio of the signal in the ischemic/non-ischemia leg; *p<0.05 vs. respective placebo treated control group).

**Supplement Figure 3. Pioglitazone does not decrease arteriogenesis.** Representative confocal image of muscle tissue stained with the smooth muscle cell marker smooth muscle actin (SMA; purple), the endothelial cell maker von Willebrand factor (vWF, red), and lectin (green) marking perfusion. Arterial density (SMA enclosed vessels/field) is increased by limb ischemia in both diabetic and non-diabetic models (*p<0.05 vs. respective placebo treated control group), but pioglitazone treatment did not significantly reduce arteriogenesis compared with vehicle control (p = 0.15).


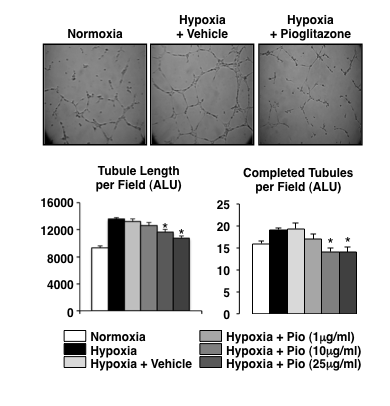


**Supplement Figure 4. Pioglitazone decreases autocrine angiogenic signaling in vitro.** Representative images hMVECs on a matrigel assay under normoxic, hypoxic, or hypoxic plus pioglitazone treatments. Pioglitazone blocks the hypoxia-induced increase in both total tubule length per field (left) and completed tubule structures per field (right) in a dose-dependent manner (n=5 images/well, 5 wells/group/experiment, 3 experiments. *p<0.05 vs. hypoxia vehicle).
